# Siglec-1-targeted nanobodies restrict HIV-1 transmission and infection of dendritic cells

**DOI:** 10.1128/jvi.00128-26

**Published:** 2026-07-29

**Authors:** Shirley Man, Alsya J. Affandi, Hendrik J. Brink, John L. van Hamme, Veronique A. L. Konijn, Joeke G. C. Stolwijk, Joke M. M. den Haan, Neeltje A. Kootstra, Teunis B. H. Geijtenbeek

**Affiliations:** 1 Department of Experimental Immunology, Amsterdam UMC Location AMC 26066, Amsterdam, the Netherlands; 2 Amsterdam Institute for Immunology and Infectious Diseases 614286 , Amsterdam, the Netherlands; 3 Department of Molecular Cell Biology and Immunology, Amsterdam UMC location VUmc 1209, Amsterdam, the Netherlands; University Hospital Tübingen, Tübingen, Germany

**Keywords:** human immunodeficiency virus, dendritic cells, nanobodies, Siglec-1, CD169

## Abstract

Human immunodeficiency virus 1 (HIV-1) remains a global health burden affecting over 39 million people worldwide, with approximately one million new infections occurring each year. Sexual transmission remains the predominant route of HIV-1 acquisition, during which the virus crosses mucosal barriers and interacts with host immune cells to facilitate infection. Dendritic cells (DCs) contribute to systemic viral spread and seeding of reservoirs by efficiently capturing HIV-1, leading to infection and subsequent transmission of the virus to CD4^+^ T cells. Siglec-1 (CD169) is a key receptor involved in HIV-1 capture, and its blockade may help prevent viral transmission. Here, we used newly developed single-domain antibodies, also known as nanobodies, against Siglec-1 as a candidate inhibitor to limit HIV-1 transmission. Using a Siglec-1-overexpressing cell line, we demonstrated that these nanobodies specifically blocked Siglec-1-mediated HIV-1 binding and transmission. Extending these findings to a more physiologically relevant context, the anti-Siglec-1 nanobodies neither induced immune nor cellular activation in DCs, indicating a favorable safety profile for functional applications. Most notably, the anti-Siglec-1 nanobody 2C2 effectively blocked HIV-1 binding as well as infection of DCs. Moreover, both replication-dependent and replication-independent HIV-1 transmission by DCs was also abrogated by the nanobody. Our findings not only underscore the relevance of Siglec-1 in HIV-1 capture but also highlight the therapeutic potential of utilizing host-targeting strategies against infectious diseases.

**IMPORTANCE:**

Sexual transmission is the main route of human immunodeficiency virus 1 (HIV-1) infection, and novel interventions are needed to prevent this crucial first step. Mucosal dendritic cells play a key role by capturing HIV-1 via attachment receptors, leading to dendritic cell infection and subsequent transmission to T cells, thereby facilitating viral spread and establishment of infection. Here, we show that small, highly specific nanobodies targeting the attachment receptor Siglec-1 strongly interfere with this early stage of HIV-1 transmission. Siglec-1 nanobodies prevented HIV-1 binding to and infection of dendritic cells, thereby blocking transmission to T cells without inducing unwanted immune activation. Together, these findings identify Siglec-1 nanobodies as promising interventions and support the development of host-directed nanobody-based strategies to reduce HIV-1 spread.

## INTRODUCTION

Human immunodeficiency virus 1 (HIV-1) continues to be a significant global health challenge with over 39 million people affected worldwide. Despite advances in diagnostic testing and established prevention campaigns, an average of 1.3 million newly acquired HIV-1 infections are still reported annually (1). Antiretroviral therapy is highly effective in suppressing HIV-1 infection, and antiretroviral drugs used as pre-exposure prophylaxis (PrEP) have proven successful in preventing HIV-1 acquisition. Moreover, novel antiretroviral drugs such as long-acting HIV-1 capsid inhibitor lenacapavir have demonstrated remarkable efficacy in preventing HIV-1 infection in clinical trials, highlighting the potential of long-acting preventive interventions (2). While these strategies act by directly targeting the virus, complementary approaches aimed at host factors involved in early viral capture and dissemination at mucosal sites may provide an additional layer of protection and further strengthen HIV-1 prevention strategies.

Sexual transmission remains the predominant route of HIV-1 acquisition worldwide, with most new infections resulting from mucosal exposure during unprotected intercourse (3). During this process, HIV-1 crosses genital or rectal mucosal barriers and engages with host immune cells to initiate systemic infection (4). Dendritic cells (DCs) continuously sample their extracellular environment for foreign antigens and, as key sentinels in mucosal tissues, are among the first to encounter invading pathogens such as HIV-1 (5, 6). HIV-1 has evolved to exploit DCs by binding specific attachment receptors and subsequently hijacking endocytic pathways as a mechanism to avoid degradation (7–9). After being captured by DCs, HIV-1 promotes the formation of virological synapses between DCs and CD4^+^ T cells for direct viral transfer while bypassing immune surveillance (10, 11). This viral transmission to target cells predominantly takes place in lymphoid tissues, where activated DCs accumulate following migration to present antigens to naïve CD4^+^ T cells (5, 11, 12). In this manner, HIV-1 transmission by DCs contributes to systemic viral spread and seeding of reservoirs.

Siglec-1 (sialic acid-binding immunoglobulin-like lectin, also known as sialoadhesin and CD169) is a membrane receptor expressed by DCs that plays a key role in viral capture and transmission (13–17). Siglec-1 is a member of the Siglec family and is thought to function primarily as an adhesion receptor, mediating cell-cell interactions and pathogen binding through preferential recognition of α2,3-linked sialic acids (18, 19). The HIV-1 envelope membrane harbors host-derived, sialic-acid-containing gangliosides that are recognized by Siglec-1-expressing DCs (16, 20, 21). These endogenous gangliosides have also been implicated in the transmission of other viruses, including Ebola virus and severe acute respiratory syndrome coronavirus (SARS-CoV)-2 (22, 23). Upon binding, Siglec-1 sequesters the virus into a specialized virus-containing compartment (VCC) that shields it from lysosomal degradation (16, 24, 25). This viral capture then allows intact virions to traffic along the viral synapse for transmission to neighboring CD4^+^ T cells. In addition to mediating viral transmission, Siglec-1 expression on DCs may increase their susceptibility to HIV-1 infection, as myeloid DC precursors that constitutively express Siglec-1 were shown to be particularly prone to infection in a Siglec-1-dependent manner (26, 27). Targeting the interaction between Siglec-1 and HIV-1 in DCs could thus represent a promising strategy to mitigate viral spread.

Nanobodies are single-domain antibody fragments derived from heavy-chain-only antibodies that retain high target affinity despite their minimal size. We previously engineered nanobodies against Siglec-1 with the capacity to inhibit receptor binding to sialic acid-containing bacterium *Campylobacter jejuni* (*C. jejuni*) and SARS-CoV-2 (28). In this study, we examined the functionality of Siglec-1-targeting nanobodies in DCs and report on their capacity to interfere with HIV-1 binding, infection, and transmission to target cells as key steps in viral dissemination and reservoir formation.

## RESULTS

### Anti-Siglec-1 nanobody specifically blocks Siglec-1-mediated binding and transmission of HIV-1

**Fig 1:**
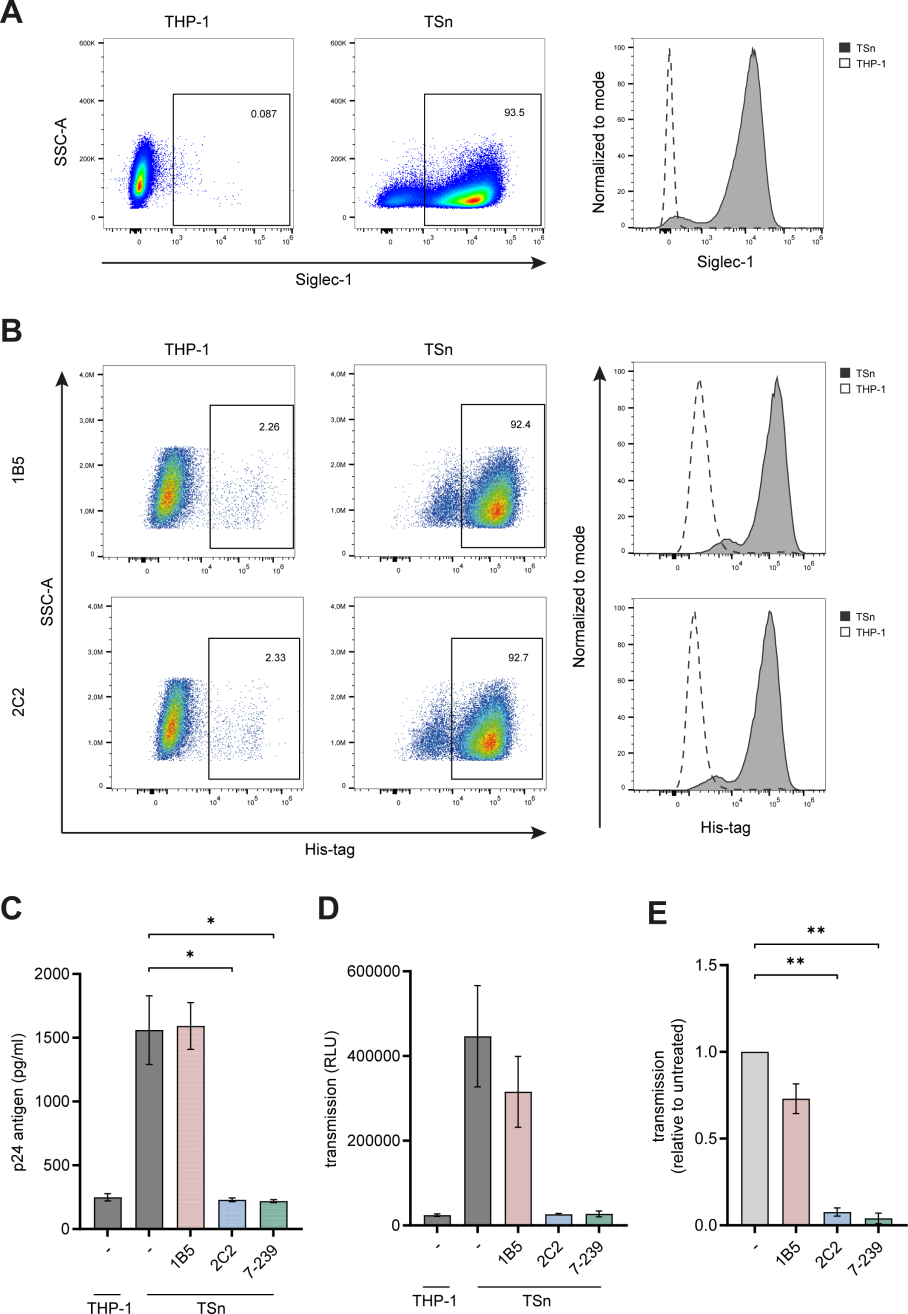
Siglec-1 nanobody 2C2 specifically blocks Siglec-1-mediated binding and transmission of HIV-1. (**A**) Representative flow cytometry plots and histogram of Siglec-1 expression on THP-1 and TSn cells. (**B**) Representative flow cytometry plots and histograms of nanobodies 1B5 or 2C2 binding to THP-1 and TSn cells. (**C**) HIV-1 uptake measured as p24 antigen (pg/mL) by TSn and THP-1 cells, which were either untreated or treated with nanobodies or 7-239 antibody. Data represent three separate experiments. (**D and E**) Infection of TZM-bl following co-culture with TSn and THP-1 cells that were either untreated or treated with nanobodies or 7-239 antibody and challenged with HIV-1. Representative (**D**) and pooled (**E**) independent experiments (*N* = 3). RLU; relative light units. Data shown are mean and SD for (**D**) and mean and SEM for (**C and E**). Statistical analysis was performed using an unpaired Welch’s t test. *P < 0.05; **P < 0.01.

We first assessed the specificity of the anti-Siglec-1 nanobodies clones 1B5 and 2C2 in targeting the receptor by using TSn cells, which are THP-1 cells transduced to constitutively express Siglec-1 (17). Expression of surface Siglec-1 was verified for TSn cells, whereas the parental THP-1 did not express the receptor (Fig. 1A). The nanobodies 1B5 and 2C2 bound to TSn cells, but not to THP-1 (Fig. 1B), indicating their specificity for the Siglec-1 receptor.

Next, we examined the capability of the anti-Siglec-1 nanobodies in blocking HIV-1 binding to Siglec-1 and compared this to the blocking antibody against Siglec-1 clone 7-239 (29, 30). Both TSn and THP-1 cells were incubated with nanobodies or antibody prior to HIV-1 exposure, and after 4 h, cells were washed extensively to measure HIV-1 binding. Siglec-1-expressing TSn cells efficiently bound HIV-1 in contrast to THP-1 cells (Fig. 1C). Nanobody 2C2 effectively blocked HIV-1 binding to TSn, while no block in binding was observed with the 1B5 nanobody.

After binding, Siglec-1 has been shown to be able to transmit HIV-1 to target cells. Therefore, we investigated whether the nanobodies were able to block HIV-1 transmission by TSn cells. Cells were exposed to HIV-1 for 4 h, followed by thorough washing to remove unbound virus. These cells were subsequently co-cultured with HIV-1 reporter cell line TZM-bl, which expresses luciferase upon HIV-1 infection. TSn efficiently transmitted HIV-1 to TZM-bl cells in contrast to mock THP-1 cells (Fig. 1D). Both the nanobody 2C2 and 7-239 antibody abrogated HIV-1 transmission by TSn cells in contrast to nanobody 1B5 (Fig. 1D and E). Collectively, these data show that the nanobodies 2C2 and 1B5 are specific for Siglec-1 and that 2C2 specifically is able to block the function of Siglec-1 in HIV-1 binding and transmission.

### Anti-Siglec-1 nanobodies do not activate moDCs

**Fig 2:**
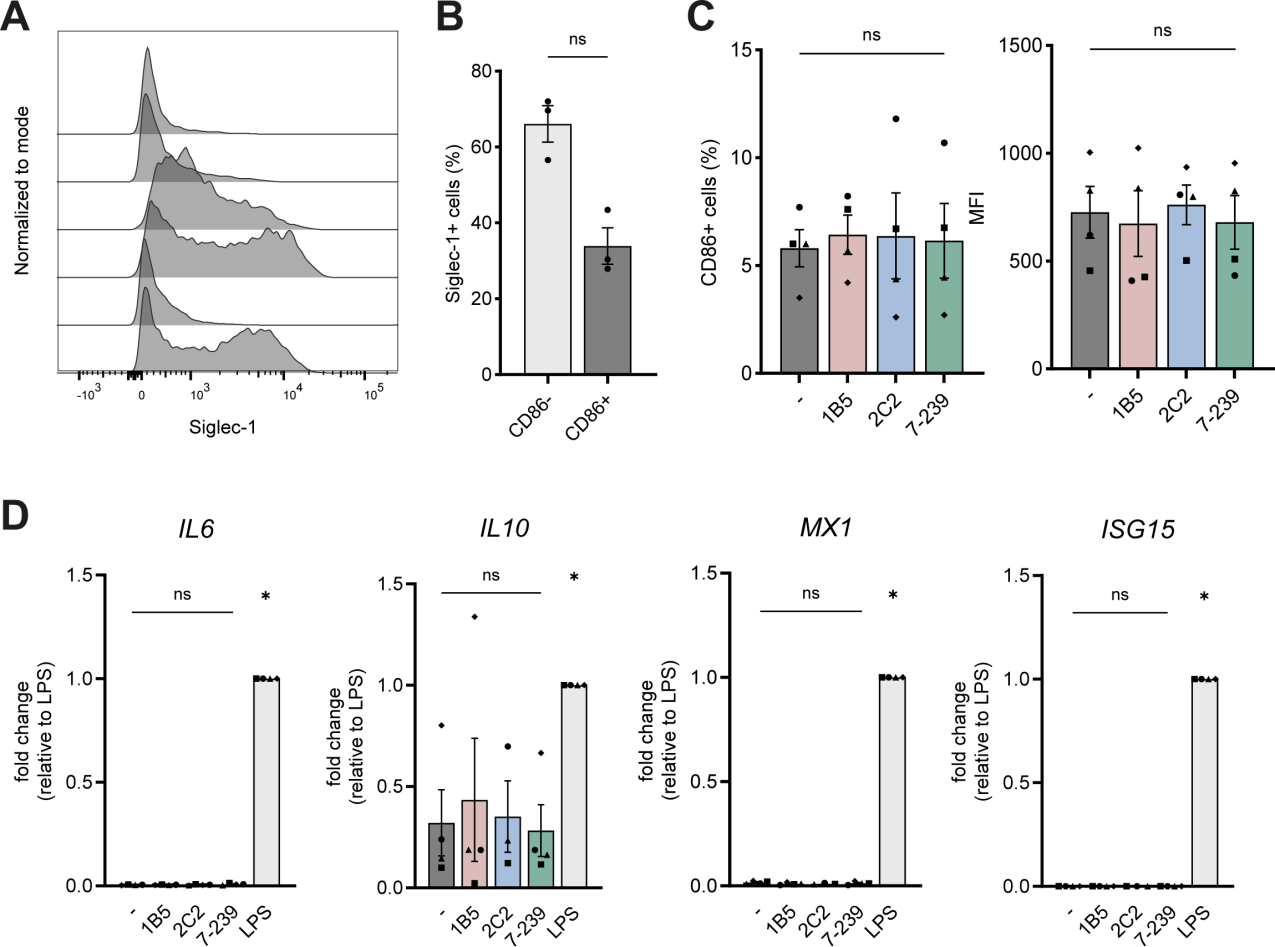
Siglec-1 nanobodies neither elicit immune responses nor induce DC activation. (**A**) Histogram overlays of Siglec-1 expression in moDCs from multiple donors (*N* = 6). (**B**) Frequency of Siglec-1^+^ cells in CD86^+^ and CD86⁻ moDC populations (*N* = 3). (**C**) Frequency and mean fluorescence intensity (MFI) of CD86^+^ cells in moDCs after 5-day incubation with either nanobodies, 7-239 antibody, or without (*N* = 4). (**D**) Gene expression in moDCs following a 6-h treatment with nanobodies, 7-239 antibody, or LPS control (*N* = 4). Fold change is normalized to *GAPDH* and relative to LPS condition. Data shown are mean and SEM. Statistical analysis was performed using a Wilcoxon signed-rank test (**B**) and Friedman test with Dunn’s *post hoc* test (**D**). ns, not significant. *P < 0.05; **P < 0.01*.*

We next determined whether the anti-Siglec-1 nanobodies induced activation of monocyte-derived DCs (moDCs) and elicited concomitant immune responses. Although Siglec-1 is expressed by DC subsets beyond moDCs, these cells are highly sensitive to activation signals and therefore well suited to investigate potential activation induced by the Siglec-1 nanobodies. Because immature moDCs from different donors displayed variable levels of Siglec-1 expression (Fig. 2A), we assessed whether this variability was related to cellular activation. Notably, most Siglec-1-expressing cells lacked CD86 expression (Fig. 2B), indicating that Siglec-1 expression did not correlate with DC activation. To test the effect of the nanobodies on DC activation, we incubated unstimulated moDCs with either the nanobodies or the 7-239 antibody for 5 days and measured the classical DC maturation marker CD86. Prolonged incubation with these agents did not lead to DC activation, as seen by the unchanged expression levels of CD86 between untreated and treated moDCs (Fig. 2C).

To assess the effect of the nanobodies on the immune response, we incubated moDCs with either the nanobodies, 7-239 antibody, or Toll-like receptor (TLR) 4 agonist lipopolysaccharide (LPS), and measured gene expression of cytokines and interferon-stimulated genes (ISGs) after 6 h. In contrast to the anti-Siglec-1 nanobodies and 7-239 antibody, only LPS induced expression of *IL6*, *IL10*, *MX1*, and *ISG15* in moDCs (Fig. 2D). The anti-Siglec-1 nanobodies did not induce cytokine or ISG expression, nor did they trigger DC maturation.

### Anti-Siglec-1 nanobody blocks HIV-1 binding to immature and IFN-α-stimulated moDCs

**Fig 3:**
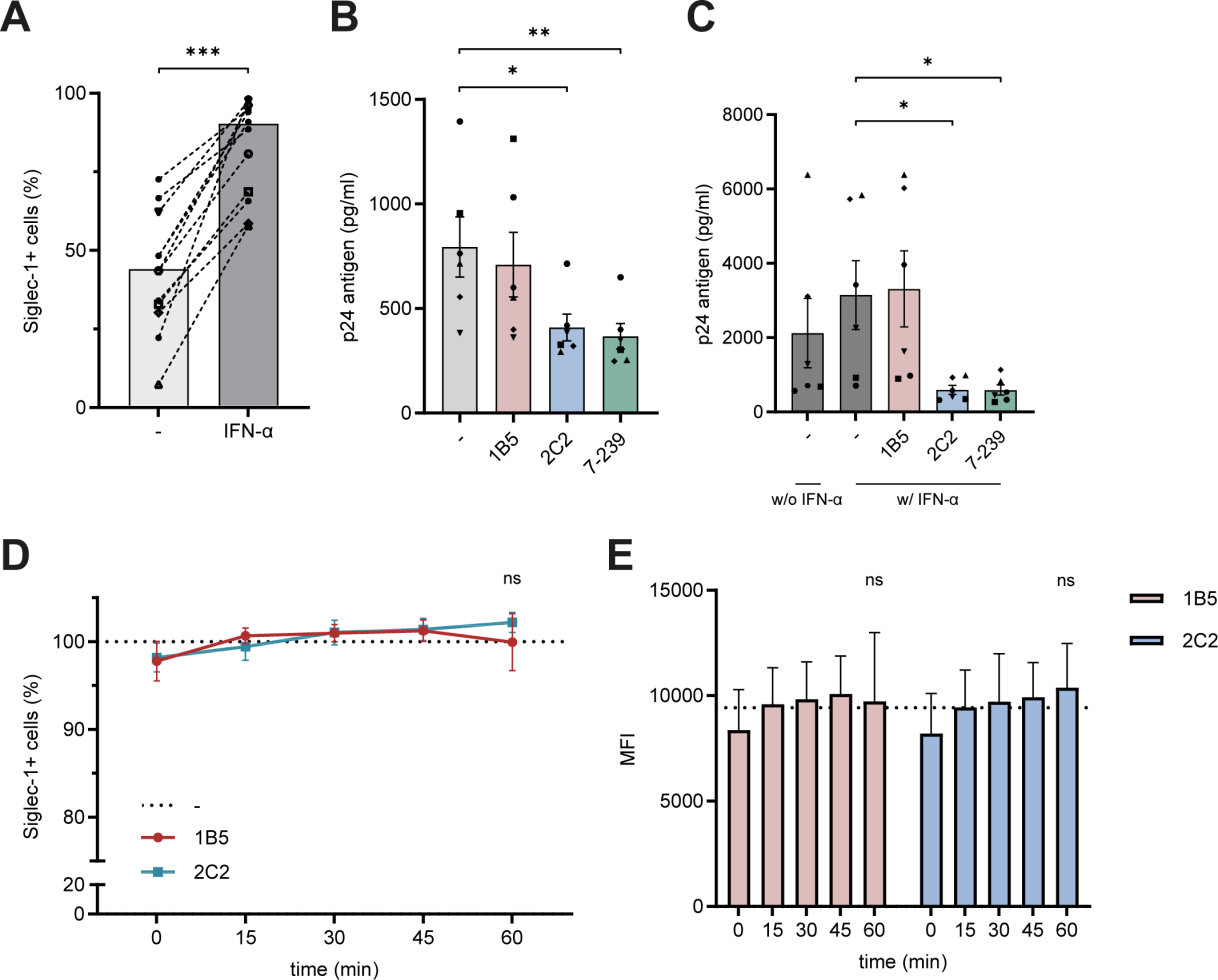
Siglec-1 nanobody 2C2 blocks HIV-1 binding to moDCs. (**A**) Siglec-1^+^ populations in unstimulated or IFN-α-stimulated moDCs from different donors (*N* = 12). (**B**) HIV-1 uptake measured as p24 antigen (pg/mL) by moDCs that were either untreated or treated with nanobodies or 7-239 antibody (*N* = 6). (**C**) Same as in panel **B**, but in moDCs pre-stimulated with IFN-α (*N* = 6). Unstimulated cells without IFN-α are included as reference. (**D and E**) Detectable surface Siglec-1 expression defined as Siglec-1^+^ cells (**D**) or mean fluorescence intensity (MFI; **E**) on IFN-α-stimulated moDCs treated with nanobodies and measured by flow cytometry at different time points (*N* = 4). The dotted line represents Siglec-1 expression in the untreated condition, set as baseline in panel **D,** and shows the MFI in panel **E**. Donors are represented by different symbols. Data shown are mean and SEM. Statistical analysis was performed using a Wilcoxon signed-rank test (**A**), paired *t*-test (**B and C**), and Friedman test with Dunn’s *post hoc* test (**D and E**). ns, not significant. *P < 0.05; **P < 0.01; ***P < 0.001*.*

We then examined whether the anti-Siglec-1 nanobody 2C2 blocked HIV-1 binding to moDCs. First, we confirmed the expression of Siglec-1 on the surface of moDCs. We detected variable levels of Siglec-1 on immature moDCs derived from healthy blood donors (Fig. 3A). Upon stimulation with IFN-α, the population of moDCs expressing Siglec-1 increased (median increase from 43% to 90%), consistent with the upregulation of the receptor as reported by others (14, 15).

Next, we pre-treated unstimulated and IFN-α-stimulated moDCs with the nanobodies or the 7-239 antibody prior to HIV-1 exposure for 4 h. moDCs from different donors bound HIV-1, albeit in a donor-dependent manner (Fig. 3B). Differences in HIV-1 capture did not correlate with Siglec-1 expression (data not shown). In contrast to 1B5, both 2C2 and the 7-239 antibody significantly reduced HIV-1 binding by moDCs from all donors. HIV-1 binding was increased following IFN-α-stimulation, and a consistently effective block in binding was observed with both the 2C2 nanobody and 7-239 antibody (Fig. 3C). To investigate whether the decreased HIV-1 binding was caused by internalization of the Siglec-1 receptor, we incubated moDCs with the nanobodies and assessed the surface expression level of Siglec-1 using a fluorophore-conjugated 7-239 antibody at different time points. Siglec-1 expression did not fluctuate from baseline after prolonged incubation with either nanobodies (Fig. 3D and E). These data strongly suggest that not only IFN-α-stimulated moDCs but also immature moDCs bind HIV-1 using Siglec-1 and that the 2C2 nanobody effectively blocks Siglec-1 interaction with HIV-1.

### Anti-Siglec-1 nanobody decreases HIV-1 infection of moDCs

**Fig 4:**
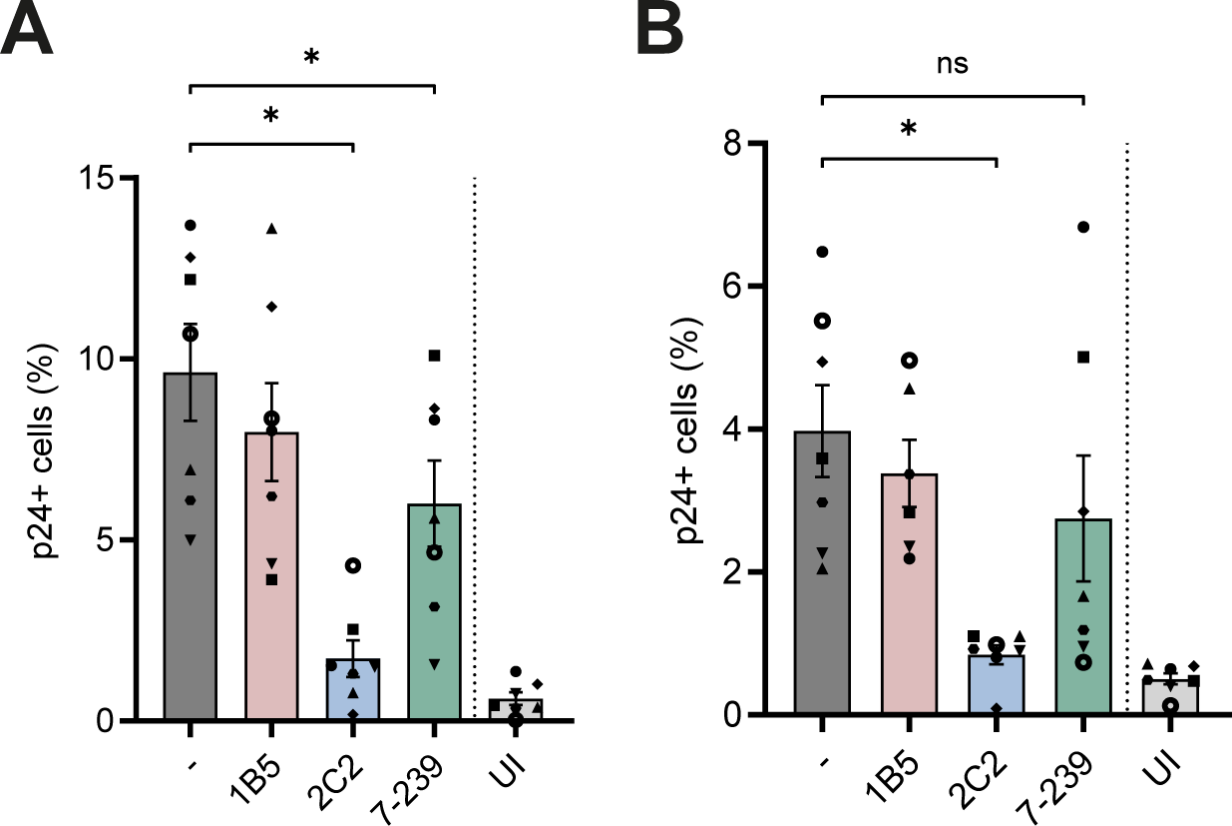
Siglec-1 nanobody 2C2 decreases HIV-1 infection of moDCs. Infection of unstimulated (**A**) and IFN-α-stimulated (**B**) moDCs that were either untreated or treated with nanobodies or 7-239 antibody and infected with HIV-1 for 5 days (*N* = 7). Donors are represented by different symbols. UI, uninfected. Data shown are mean and SEM. Statistical analysis was performed using a paired Wilcoxon signed-rank test. ns, not significant. *P < 0.05*.*

Although Siglec-1 has recently been shown to be involved in infection of precursor DCs (26), most studies have primarily focused on Siglec-1 in moDCs following IFN-α- or LPS-treatment, conditions that render these cells largely refractory to HIV-1 infection (31–34). Here, we examined more closely the relevance of Siglec-1 in HIV-1 infection of unstimulated DCs by exposing the cells to R5-tropic HIV-1 for 5 days. moDCs from all donors were infected at varying levels as measured by the presence of p24^+^ cells (Fig. 4A). Notably, treatment of moDCs with the 2C2 nanobody strongly blocked HIV-1 infection, whereas no significant inhibition was observed with 1B5. Similarly, the 7-239 antibody also blocked HIV-1 infection of moDCs. Consistent with the antiviral effect of IFN-α, exposure of IFN-α-stimulated moDCs to HIV-1 resulted in lowered infection (Fig. 4B). While the 7-239 antibody did not reduce infection levels further, the 2C2 nanobody markedly blocked HIV-1 infection. These findings suggest that Siglec-1 plays a role in fostering HIV-1 infection of DCs and that the anti-Siglec-1 nanobody 2C2 can strongly reduce the susceptibility of DCs to infection.

### Anti-Siglec-1 nanobody blocks HIV-1 transmission by moDCs

**Fig 5:**
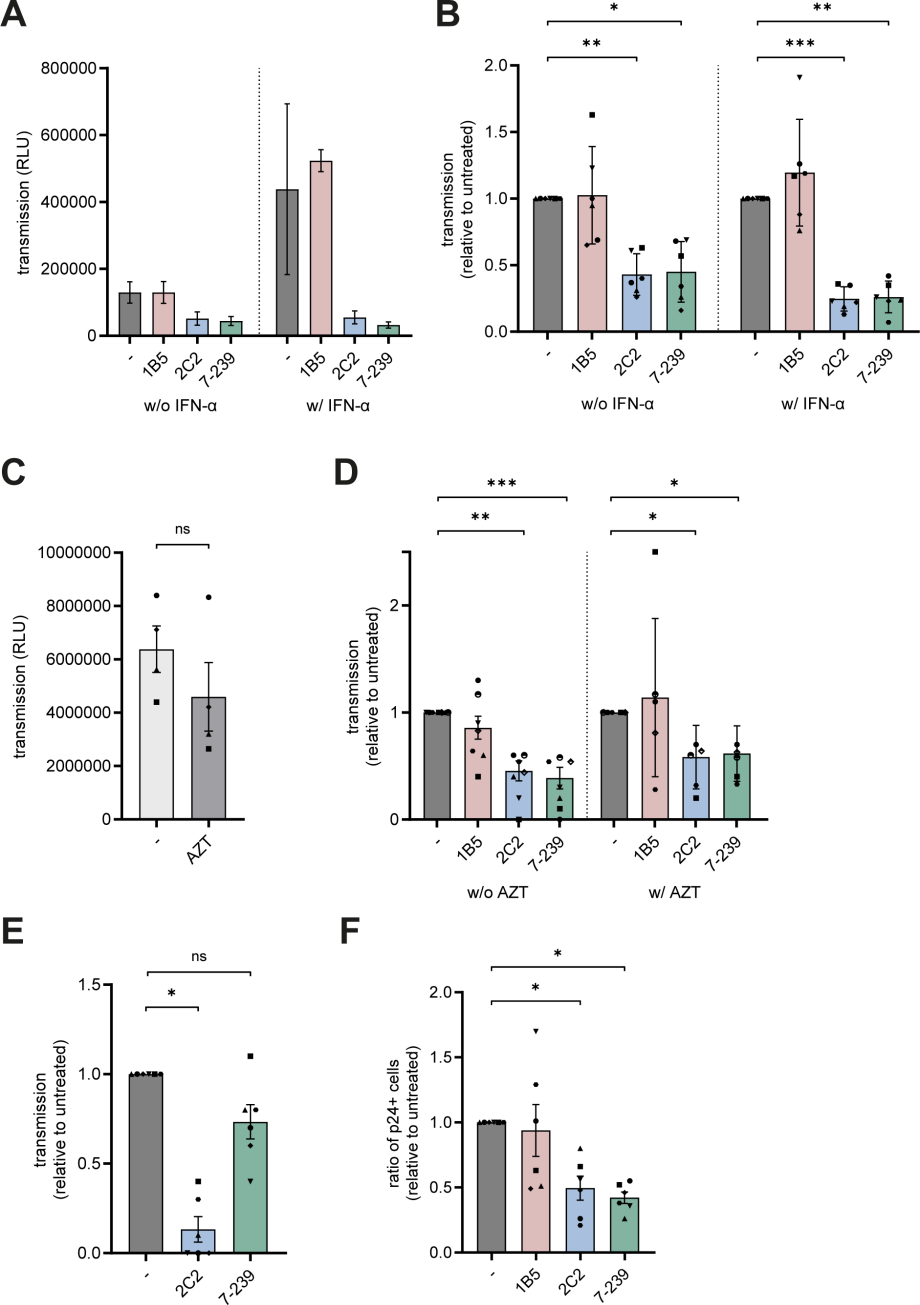
Siglec-1 nanobody 2C2 diminishes HIV-1 transmission by moDCs. (**A and B**) Infection of TZM-bl following co-culture with unstimulated or IFN-α-stimulated moDCs that were either untreated or pre-treated with nanobodies or 7-239 antibody and challenged with HIV-1 for 4 h (*N* = 6). Representative (**A**) and pooled (**B**) independent experiments. (**C**) Infection of TZM-bl following co-culture with unstimulated moDCs in the presence or absence of AZT and challenged with HIV-1 for 48 h (*N* = 4). (**D**) Infection of TZM-bl following co-culture with unstimulated moDCs that were either untreated or treated with nanobodies or 7-239 antibody, in the presence or absence of AZT (*N* = 8 and 6, respectively), and challenged with HIV-1 for 48 h. (**E**) Infection of TZM-bl following co-culture with unstimulated moDCs that were challenged with HIV-1 for 48 h in the presence of AZT and afterward either left untreated or treated with 2C2 nanobody or 7-239 antibody (*N* = 6). (**F**) Infection of autologous CD4 T cells following co-culture with IFN-α-stimulated moDCs that were either untreated or pre-treated with nanobodies or 7-239 antibody and challenged with HIV-1 for 4 h (*N* = 6). Donors are represented by different symbols. RLU; relative light units. Data are mean and SD for (**A**) and mean and SEM for (**B–F**). Statistical analysis was performed using a ratio paired t test (**B–D**), and a paired Wilcoxon signed-rank test (**E and F**). ns, not significant. *P < 0.05; **P < 0.01; ***P < 0.001*.*

Finally, we assessed the capacity of the anti-Siglec-1 nanobodies in blocking HIV-1 transmission to target cells by moDCs. Both immature and IFN-α-stimulated moDCs were exposed to HIV-1 for 4 h and after extensive washing were subsequently co-cultured with TZM-bl cells. Transmission was determined by measuring luciferase activity after 2 days. We observed higher levels of HIV-1 transmission for IFN-α-stimulated compared to immature moDCs (Fig. 5A). Transmission was strongly reduced when cells were pre-treated with either the 2C2 nanobody or 7-239 antibody (Fig. 5A and B). In contrast, varied levels of viral transmission were observed with nanobody 1B5, consistent with its variable effects on HIV-1 binding.

Since prolonged virus exposure increases the risk of HIV-1 infection in DCs (9), we evaluated the contribution of Siglec-1 in facilitating HIV-1 transmission both in the presence and absence of viral replication. Unstimulated moDCs were exposed to HIV-1 for 48 h, and zidovudine (AZT) was used to block virus infection at the step of reverse transcription. Following extensive washing to remove unbound virus, cells were co-cultured with TZM-bl to compare transmission levels between AZT-treated and untreated moDCs. HIV-1 was efficiently transmitted by moDCs to TZM-bl cells as determined by luciferase activity, and AZT did not significantly influence transmission levels (Fig. 5C). These findings suggest that *de novo* virion production by moDCs after extended viral exposure is not required for HIV-1 transmission. Importantly, upon prior incubation with nanobody 2C2 and antibody 7-239, HIV-1 transmission to TZM-bl was reduced by twofold for both AZT-treated and untreated moDCs (Fig. 5D).

HIV-1 is internalized into VCCs, which remain connected to the extracellular space (35). Given the small size of the nanobody, we investigated whether 2C2 is able to block transmission after HIV-1 exposure. Immature DCs were first challenged with virus in the presence of AZT, extensively washed, and subsequently incubated with the 2C2 nanobody or the 7-239 antibody before co-culture with TZM-bl cells. Notably, 2C2, but not 7-239, blocked HIV-1 transmission under these conditions (Fig. 5E), suggesting that its small size enables access to VCCs and thereby prevents subsequent transmission.

Finally, to assess the effect of the nanobodies in a more physiologically relevant target cell model, HIV-1 transmission from DCs to autologous CD4 T cells was evaluated. To ensure proper targeting in this system, moDCs were first stimulated with IFN-α to induce Siglec-1 expression, providing a consistent and robust experimental setup for the assay. Next, moDCs were pre-incubated with the nanobodies or the 7-239 antibody before challenge with HIV-1. After 4 h, cells were extensively washed and transferred to T cells for co-culture. AZT was added to limit secondary rounds of infection, and T cell infection was quantified by intracellular p24 levels after 5–7 days. Both 2C2 and 7-239 reduced the number of infected T cells compared to the untreated condition (Fig. 5F). These data indicate that the 2C2 nanobody effectively blocks Siglec-1-mediated HIV-1 transmission from DCs to T cells. Overall, these findings are concordant with our previous observations, reinforcing the potential of nanobody 2C2 as a targeted strategy to interrupt Siglec-1-dependent HIV-1 dissemination by DCs.

## DISCUSSION

DCs play a critical role in HIV-1 dissemination by facilitating viral transmission to long-lived CD4^+^ T cells. Siglec-1 has been shown to be an important HIV-1 attachment receptor on DCs, and blocking this receptor could therefore prove to be beneficial as an antiviral strategy. In this study, we employed nanobodies targeting Siglec-1 and observed a robust inhibition of virus binding by Siglec-1^+^ cells, reduced infection of moDCs, and diminished transmission of HIV-1 to target cells.

We noted a strong block in HIV-1 binding to Siglec-1 upon treatment with the Siglec-1-specific 2C2 nanobody. Since Siglec-1 remained detectable on the cell surface, the reduced HIV-1 binding likely reflects receptor masking rather than receptor internalization. Siglec-1 binds sialic acids via a conserved binding pocket in its extracellular V-set domain, and mutations of conserved residues, such as R116A, effectively abolish HIV-1 binding (14, 36–38). As shown in our previous study, the 2C2 nanobody clone binds specifically to the ligand-binding site of Siglec-1 (28). Displacement assays further demonstrated that 1B5 binds to a distinct site from 2C2 (28), which likely explains its lack of inhibitory activity against HIV-1. In contrast, by occupying the essential sialic acid-binding pocket, nanobody 2C2 effectively prevents Siglec-1 from engaging HIV-1.

While Siglec-1 is not known to signal through classical immunoreceptor tyrosine-based inhibitory motifs, localized signaling cannot be entirely dismissed. This is exemplified by the formation of VCCs upon HIV-1 binding (35). As these structures remain connected to the extracellular space, they do not constitute classical endocytic compartments. Rather, their formation involves membrane remodeling and invagination likely driven by actin cytoskeletal rearrangements following Siglec-1 clustering (25, 39), suggesting that receptor engagement can induce localized cellular responses. In addition, suppression of innate immune responses via Siglec-1 has been reported in macrophages, suggesting a potential context-dependent signaling capacity (40, 41). Our data indicate that the 2C2 nanobody did not trigger such signaling events in moDCs. During prolonged incubation, no changes in activation markers or in the expression of cytokines and antiviral ISGs were observed. Moreover, we did not detect internalization of the Siglec-1 receptor within the time frame tested. Collectively, these findings indicate that 2C2 does not induce measurable immune or cellular activation in moDCs, supporting its suitability as a blocking agent.

Despite its known interaction with HIV-1, the role of Siglec-1 in HIV-1 infection of DCs has remained poorly defined, in part because prior studies primarily employed IFN-α- or LPS-treated moDCs that express higher levels of the receptor (14, 16). Here, we found that blocking Siglec-1 reduced HIV-1 infection levels in immature moDCs, suggesting a role for the receptor in promoting infection. This is in line with the finding that constitutive Siglec-1-expressing precursor DCs are uniquely susceptible to HIV-1 infection compared to other blood DC subsets, which was shown to be independent of HIV-1 co-receptor expression (26). Moreover, Siglec-1 expression has been associated with increased HIV-1 infection in primary monocyte-derived macrophages, and receptor blockade was found to inhibit infection of these cells (42, 43). The direct binding of HIV-1 to Siglec-1 likely enhances the susceptibility of expressing cells by facilitating prolonged interactions with virions. However, whether Siglec-1 promotes infection as an attachment receptor by positioning virus in closer proximity to HIV-1 entry receptors at the membrane to allow fusion, or through alternative mechanisms, remains to be elucidated. Strikingly, we observed that nanobody 2C2 blocked infection of DCs more effectively than the 7-239 antibody, despite both compounds inhibiting HIV-1 capture. The different experimental time scales, namely, 4 h for capture versus 5 days for infection, may have an influence on their modulatory effects. Based on previous work, 2C2 likely binds a distinct epitope on Siglec-1 compared to 7-239 (28), and it is therefore possible that 2C2 exerts additional downstream effects that enhance its ability to inhibit HIV-1 infection. Notably, 2C2, in contrast to 7-239, also blocked HIV-1 transmission after viral exposure. This suggests that the nanobody can access VCCs and interfere with Siglec-1-mediated HIV-1 transfer, which may underlie its enhanced inhibition of infection.

HIV-1 attachment blockers may offer a complementary strategy in settings where PrEP adherence is suboptimal, particularly when formulated for topical or long-acting delivery to provide protection at mucosal sites of viral exposure, thereby reducing reliance on frequent dosing and adherence. By targeting host factors involved in early viral attachment, such approaches are also less influenced by viral subtype diversity. Early events during transmission remain largely insensitive to current antiretroviral strategies, with DC-mediated infection of T cells persisting even in the presence of tenofovir and raltegravir (44). Blocking transmission is therefore critical to limit further HIV-1 spread and reservoir seeding. Our findings in moDCs demonstrate that nanobodies targeting Siglec-1 not only effectively block HIV-1 binding but also ultimately restrict transmission to target cells, providing proof-of-principle that interference with Siglec-1-mediated virus capture may serve as a preventive strategy at early stages of infection. As variation in HIV-1 transmission efficiencies and Siglec-1-targeted blockade were observed, inter-individual expression levels of Siglec-1 and additional attachment receptors should be considered (8, 45, 46). Combined nanobody targeting of these receptors may therefore be required for more complete inhibition of transmission.

These early transmission events extend to tissue compartments, as Siglec-1^+^ DCs readily capture HIV-1, facilitating its dissemination to lymphoid tissues where productive DC-T cell interactions support viral persistence (11, 47). In addition, Siglec-1^+^ tissue-resident macrophages may act as long-lived viral reservoirs via the formation of VCCs (48). These observations underscore the need for therapeutics that are effective across relevant tissue environments. In this context, nanobodies offer several advantages over conventional antibodies. Their smaller size enables improved tissue penetration and access to sterically restricted epitopes, making them particularly well suited to interfere with virus-host interactions (49). Their single-domain structure further facilitates engineering into multi-specific formats and fusion constructs, allowing flexible modulation of affinity, valency, and effector function. The half-life of nanobodies can also be improved through such engineering, for example, by fusion with albumin-binding or human Fc domains (50). The observation that 2C2, but not the 7-239 antibody, effectively blocked HIV-1 transmission to target cells after viral exposure suggests that the nanobody can access VCCs to inhibit Siglec-1-mediated viral transfer, potentially due to its smaller size. Nanobody clone 2C2 could therefore be a valuable agent in limiting HIV-1 transmission. Further investigation is warranted to evaluate its *in vivo* distribution and functional efficacy in diverse anatomical sites. As loss-of-function or null variants in Siglec-1 have been described in individuals without overt immunodeficiency (51), partial or localized inhibition may be tolerated. However, given that Siglec-1 on sentinel macrophages is also known to promote antiviral CD8^+^ T cell responses (52, 53), systemic blockade may be undesirable. These considerations suggest that the 2C2 nanobody may be best applied in localized or DC-targeted approaches to limit HIV-1 transmission while preserving systemic immune function.

In addition to blocking HIV-1, we have previously shown that the 2C2 nanobody can inhibit Siglec-1 binding to *C. jejuni* and SARS-CoV-2 (28). While the downstream effects of anti-Siglec-1 nanobody interference in these contexts remain to be determined, our findings suggest broader utility against other sialic acid-containing pathogens. In parallel, monoclonal antibodies targeting Siglec-1 have recently been optimized and were shown to have broad antiviral activity against ganglioside-containing enveloped viruses *in vitro* (54). Together, these results support the potential of anti-Siglec-1 therapies as a pan-pathogen strategy to counter diverse infectious agents that exploit this receptor. We anticipate that anti-Siglec-1 nanobodies may be further expanded for use against a wider array of enveloped pathogens.

In conclusion, we demonstrate that Siglec-1-targeted nanobodies can exert protective effects against HIV-1 by limiting virus-DC interactions. Host factor-targeted therapeutics represent a complementary strategy to current antiretroviral treatments, and nanobodies, due to their small size and specificity, are a promising novel tool that can be harnessed to curtail infection. Our findings support the further development of anti-Siglec-1 nanobodies as candidate therapeutics to block HIV-1 transmission and dissemination, and underscore the broader potential of nanobody-based modulation of host factors in antiviral strategies.

## MATERIALS AND METHODS

### Primary monocyte-derived dendritic cells and T cells

Human monocytes were isolated from buffy coats of healthy volunteer blood bank donors (Sanquin, Amsterdam, the Netherlands) and differentiated into monocyte‐derived DCs. Briefly, peripheral blood mononuclear cells (PBMCs) were isolated by density gradient centrifugation using Lymphoprep (Axis Shield, Dundee, UK). Subsequently, monocytes were collected after Percoll (Cytiva, Delaware, DE, USA) gradient centrifugation. Monocytes were cultured in RPMI 1640 (Gibco) supplemented with 10% fetal calf serum (FCS; Cytiva), L‐glutamine (2 mM; Lonza, Basel, Switzerland), penicillin (100 U/mL; Invitrogen, Carlsbad, CA, USA) and streptomycin (100 μg/mL; Invitrogen) in the presence of GM‐CSF (800 U/mL; Miltenyi Biotec, Bergisch Gladbach, Germany), and IL‐4 (500 U/mL; Miltenyi Biotec) at 37°C and 5% CO_2_ for 6 days to obtain monocyte‐derived DCs (moDCs). Where indicated, moDCs were treated with IFN-α (100 U/mL) for 48 h to induce upregulated expression of Siglec-1. CD4 T cells were isolated from PBMCs using the Memory CD4^+^ T Cell Isolation Kit following manufacturer’s instructions (Miltenyi Biotec). Cells were subsequently activated with anti-CD3 and anti-CD28 antibodies for 48 h prior to co-culture.

### Cell lines

THP-1 and Siglec-1-expressing THP-1 cells (THP-1-Sialoadhesin, TSn) (17) were cultured in RPMI 1640 (Gibco) supplemented with 10% FCS and antibiotics (100 U/mL penicillin, 100 μg/mL streptomycin), and maintained at 37°C and 5% CO_2_ (17). TZM-bl cells (CVCL_B478) were cultured in Iscove’s Modified Dulbecco’s Medium (Gibco) supplemented with 10% FCS and antibiotics (100 U/mL penicillin, 100 μg/mL streptomycin) and maintained at 37°C and 10% CO_2_.

### Siglec-1 nanobody production

Nanobodies against Siglec-1 were generated as described previously (28). In brief, llama immunization was performed using IFN-α-treated moDCs, TSn cells, or recombinant human Siglec-1 protein by QVQ Holding B.V. (Utrecht, The Netherlands). Subsequently, B cell isolation, complementary DNA (cDNA) library preparation, initial clone selection, and nanobody production were performed. The characterization of these nanobodies, including binding specificity, affinity measurements, and IC₅₀ determination, has been described previously (28).

### Nanobody and antibody treatment

THP-1, TSn cells, or moDCs were treated with nanobodies 1B5, 2C2 (both 500 nM), or Siglec-1 antibody 7-239 (10 µg/mL; produced in-house) (29) for 30 min at room temperature prior to virus exposure. Where indicated, nanobodies and antibody were added after viral exposure and incubated for 30 min at room temperature prior to co-culture with target cells. For the assessment of Siglec-1 internalization, moDCs were first pre-incubated with the nanobodies at 4°C for 15 min. Cells were then washed, and medium was replaced before incubation at 37°C for various time points. To determine the induction of cytokines in moDCs, cells were treated with the nanobodies, 7-239 antibody, or LPS (10 ng/mL; Sigma-Aldrich, Burlington, MA, USA) and harvested after 6 h.

### Reverse transcription quantitative PCR (RT-qPCR)

Cells were lysed and mRNA was extracted using the mRNA Catcher PLUS purification kit (ThermoFisher Scientific, Waltham, MA, USA). cDNA was synthesized using a reverse transcriptase kit (Promega, Madison, WI, USA), and quantitative PCR amplification was performed with SYBR green (ThermoFisher Scientific) using the 7500 Fast Real‐Time PCR System (Applied Biosystems, Waltham, MA, USA). The normalized amount of target mRNA was calculated from the Ct values obtained for both target and household GAPDH mRNA with the equation Nt = 2 ^Ct (GAPDH)‐Ct(target)^. The following primers were used and designed with Primer Express Software v2.0 (Applied Biosystems): GAPDH, forward primer (CCATGTTCGTCATGGGTGTG), reverse primer (GGTGCTAAGCAGTTGGTGGTG). *IL6*, forward primer (TGCAATAACCACCCCTGACC), reverse primer (TGCGCAGAATGAGATGAGTTG). *IL10*, forward primer (GAGGCTACGGCGCTGTCAT), reverse primer (CCACGGCCTTGCTCTTGTT). *MX1*, forward primer (TTCAGCACCTGATGGCCTATC), reverse primer (GTACGTCTGGAGCATGAAGAACTG). *ISG15*, forward primer (TTTGCCAGTACAGGAGCTTGTG), reverse primer (GGGTGATCTGCGCCTTCA).

### Virus production

HIV-1 NL4-3 BaL was obtained through the NIH HIV reagent program. HIV-1 NL4-3 BaL was produced by transient transfection of HEK293T cells with NL4-3 BaL plasmids (10 µg) using the calcium phosphate method. Briefly, plasmid DNA was diluted in 0.042 M HEPES containing 0.15 M CaCl_2_, subsequently mixed with an equal volume of 2× HEPES buffered saline pH 7.2, incubated at room temperature for 15 min, and added to the culture medium. After 24 h incubation in a humidified 3% CO_2_ incubator at 37°C, the culture medium was replaced, and cultures were continued at 10% CO_2_ at 37°C. Virus was harvested at 48 and 72 h after transfection and passed through a 0.22 μm filter. HIV-1 virus titers were quantified by determining the tissue culture infectious dose (TCID_50_) on TZM-bl. The amount of HIV‐1 capsid protein p24 was quantified by p24 antigen enzyme‐linked immunosorbent assay (ELISA; ZeptoMetrix, Buffalo, NY, USA)

### Virus binding assay

THP-1, TSn cells, or primary moDCs were challenged with virus (134 pg p24) for 4 h at 37°C. Subsequently, cells were washed extensively to remove any unbound virus before lysis with 0.1% Triton X-100. Bound virus was measured as p24 antigen levels using RETRO-TEK HIV-1 p24 Antigen ELISA 2.0 (ZeptoMatrix) according to the manufacturer’s protocol.

### Virus transmission assay

THP-1, TSn cells, or primary moDCs were challenged with virus (101 pg p24) for 4 or 48 h at 37°C. To investigate the role of infection and *de novo* virus production in transmission, reverse transcriptase inhibitor zidovudine (AZT, 10 µM) was used to block virus production during the 48 h incubation. Cells were then washed extensively to remove unbound virus and subsequently co-cultured with TZM-bl cells for 2 or 3 days. TZM-bl cells were pre-treated with HIV-1 protease inhibitor indinavir (IDV, 1 µM) for single-round infections. HIV-1 transmission was quantified by LTR-driven luciferase activity from TZM-bl using an in-house luciferase activity reagent substrate (0.83 mM ATP, 0.83 mM of d-Luciferin (Duchefa Biochemie B.V., Haarlem, The Netherlands), 18.7 mM MgCl_2_, 0.78 µM Na_2_H_2_P_2_O_7_, 38.9 mM Tris (pH 7.8), 0.39% glycerol, 0.03% Triton X-100, and 2.6 μM dithiothreitol). Luminescence was measured using a luminometer (Berthold Technologies, Bad Wildbad, Germany) and expressed as relative light units (RLU). For transmission to autologous CD4 T cells, DCs were challenged with HIV-1 for 4 h, followed by extensive washing to remove unbound virus. DCs were then transferred to activated CD4 T cells for co-culture. After 2 and 5 days, medium was refreshed, and AZT (10 µM) was added to the cells to block further rounds of infection. After 5–7 days, cells were harvested and infection was assessed by intracellular anti-p24 staining (see below).

### Virus infection assay

Primary moDCs were inoculated with virus (2.8 × 10⁵ TCID_₅₀_ per 10⁶ cells) for 5 days at 37°C, with medium refreshed after 2 days. Cells were subsequently harvested, and infection levels were assessed by intracellular anti-p24 staining (see below).

### Flow cytometry

Anti-human antibodies CD86 (2331-PE; BD Biosciences, New Jersey, NJ, USA) and Siglec-1 (7-239-Alexa Fluor 647; BioLegend, California, CA, USA) were used to assess surface receptor expression. For intracellular p24 staining, cells were first fixed using 4% (wt/vol) paraformaldehyde, followed by permeabilization with phosphate buffered saline (PBS) supplemented with 0.5% saponin (Sigma-Aldrich) and 0.5% bovine serum albumin (BSA) for 10 min. Cells were then stained with anti-HIV-1 p24 core antigen antibody (KC57-FITC; Beckman Coulter, Brea, CA, USA) in permeabilization buffer. For transmission experiments with autologous CD4 T cells, anti-human CD3 antibody (UCHT1-APC/Fire 750; BioLegend) was used for gating of T cells. Samples were acquired on a BD FACS Canto II and Fortessa (BD Biosciences), and data were analyzed using FlowJo software version 10 (TreeStar, Ashland, OR, USA).

### Statistical analysis

Fold differences were calculated by subtracting background signals from negative controls and expressed relative to untreated conditions. Statistical analyses were performed on background-subtracted values using GraphPad Prism software version 10.2 (GraphPad Software Inc., San Diego, CA, USA). Differences between conditions were determined by two-tailed Wilcoxon signed-rank test, Friedman test (with Dunn’s *post hoc* test), and *t*-tests for unpaired and paired observations (with Welch’s correction). Where indicated, a paired ratio *t*-test was used. Statistical significance was set at *P* < 0.05.

## Data Availability

The data supporting the findings of this study are available within the article.
